# Revisiting Cell Death Responses in Fibrotic Lung Disease: Crosstalk between Structured and Non-Structured Cells

**DOI:** 10.3390/diagnostics10070504

**Published:** 2020-07-21

**Authors:** Kiyoharu Fukushima, Takashi Satoh, Hiroshi Kida, Atsushi Kumanogoh

**Affiliations:** 1 Department of Respiratory Medicine and Clinical Immunology, Osaka University Graduate School of Medicine, 2-2 Yamadaoka, Suita, Osaka 565-0871, Japan; kumanogo@imed3.med.osaka-u.ac.jp; 2 Department of Host Defense, Research Institute for Microbial Diseases (RIMD), Osaka University, 3-1 Yamadaoka, Suita, Osaka 565-0871, Japan; sohsatoh@biken.osaka-u.ac.jp; 3 Laboratory of Host Defense, World Premier Institute Immunology Frontier Research Center (WPI-IFReC), Osaka University, 3-1 Yamadaoka, Suita, Osaka 565-0871, Japan; 4 Department of Respiratory Medicine, National Hospital Organization Osaka Toneyama Medical Centre, 5-1-1 Toneyama, Toyonaka, Osaka 560-8552, Japan; hiroshi.kida@icloud.com

**Keywords:** fibrosis, RBM7, SatM, apoptosis, macrophage, natural autoantibody

## Abstract

Fibrosis is a life-threatening disorder caused by excessive formation of connective tissue that can affect several critical organs. Innate immune cells are involved in the development of various disorders, including lung fibrosis. To date, several hematopoietic cell types have been implicated in fibrosis, including pro-fibrotic monocytes like fibrocytes and segregated-nucleus-containing atypical monocytes (SatMs), but the precise cellular and molecular mechanisms underlying its development remain unclear. Repetitive injury and subsequent cell death response are triggering events for lung fibrosis development. Crosstalk between lung structured and non-structured cells is known to regulate the key molecular event. We recently reported that RNA-binding motif protein 7 (RBM7) expression is highly upregulated in the fibrotic lung and plays fundamental roles in fibrosis development. RBM7 regulates nuclear degradation of NEAT1 non-coding RNA, resulting in sustained apoptosis in the lung epithelium and fibrosis. Apoptotic epithelial cells produce CXCL12, which leads to the recruitment of pro-fibrotic monocytes. Apoptosis is also the main source of autoantigens. Recent studies have revealed important functions for natural autoantibodies that react with specific sets of self-antigens and are unique to individual diseases. Here, we review recent insights into lung fibrosis development in association with crosstalk between structured cells like lung epithelial cells and non-structured cells like migrating immune cells, and discuss their relevance to acquired immunity through natural autoantibody production.

## 1. Introduction

Fibrosis is a chronic progressive disorder that causes severe damage to several critical organs and can lead to life-threatening tissue dysfunction through excessive deposition of the extracellular matrix (ECM). Pulmonary fibrosis is the terminal stage of a broad range of heterogeneous interstitial lung diseases (ILDs) that can be divided into various disease types [1].

Idiopathic pulmonary fibrosis (IPF) is the most common progressive ILD of unknown origin. Its prognosis is very poor, with a median survival of three–five years after diagnosis [2]. IPF is characterized by distinct histopathological and radiological findings of usual interstitial pneumonia of unknown cause. Although the pathogenetic mechanisms remain to be determined, it has long been believed that a chronic inflammatory process holds the key to fibrosis development (inflammation hypothesis) [3]. In this hypothesis, chronic inflammation injures and damages the lung and provokes fibrogenesis, leading to the development of fibrosis. Based on this model, anti-inflammatory agents such as corticosteroids and immunosuppressive agents have gained rationales for use and are regarded as the main promising treatment methods. However, despite considerable efforts to examine the impacts of inflammation on the fibrotic disease process through clinical measurements of histopathological, radiological, and serum inflammatory markers, there still exists little evidence that inflammation is prominent in the disease process of fibrotic lung diseases such as IPF [4]. Moreover, clinical trials on the impact of anti-inflammatory therapy have been performed. For example, the PANTHER IPF clinical trial was conducted to assess the impact of a potent anti-inflammatory regimen of prednisone, azathioprine, and N-acetylcysteine [5]. However, this randomized, double-blind, placebo-controlled trial found that mortality and risk of hospitalization were increased in patients treated with the potent anti-inflammatory regimen compared with the placebo, suggesting that inflammation may not be an essential component of fibrosis development in IPF. These observations challenge the hypothesis that inflammation itself drives fibrogenesis, and thus research interests have shifted toward the identification of other essential processes that can lead to lung fibrosis [4,6]. Currently, the progressive fibrotic reactions in fibrosis are considered to reflect an aberrant wound healing process, and clarification of the cascades specific to fibrogenesis is the main issue in fibrosis research. We recently investigated the mechanism of fibrosis onset and development in the lung [7,8]. In our previous studies, we found that RNA-binding motif protein 7 (RBM7) in lung structured cells is selectively elevated in the fibrotic phase and critical for fibrosis development, and uncovered an unprecedented role of sustained cell death of lung structured cells in the fibrotic phase regulated by RBM7 via nuclear degradation of NEAT1 non-coding RNA (ncRNA). Further, apoptotic lung epithelial cells in the fibrotic lung produce CXCL12, which leads to the recruitment of pro-fibrotic monocytes, i.e., segregated-nucleus-containing atypical monocytes (SatMs), resulting in the initiation of fibrosis. 

Taken together, these results suggest that close interactions between structured cells like lung epithelial cells and non-structured cells like migrated immune cells are critical for myofibroblastdifferentiation/activation leading to fibrosis development.

Failure of central or peripheral immunological tolerance leads to the production of autoantibodies, which are generated against self-antigens [9]. However, recent studies have revealed that sera from patients both with and without autoimmune diseases contain considerable amounts of IgG subclass autoantibodies that react with disease-specific sets of self-antigens [10,11]. After disease onset, tissue damage is induced and requires an effective debris clearance system, leading to increased production of natural autoantibodies [10]. Therefore, natural autoantibodies are thought to be produced for clearance of debris as an adaptive mechanism. In addition, certain disease environments can induce relocation of particular antigens to the cell surface. Thus, ongoing disease environments may be reflected by disease-specific natural autoantibodies, which can potentially modify the original disease course through associations with extracellular or cell surface proteins [12]. 

**Figure 1:**
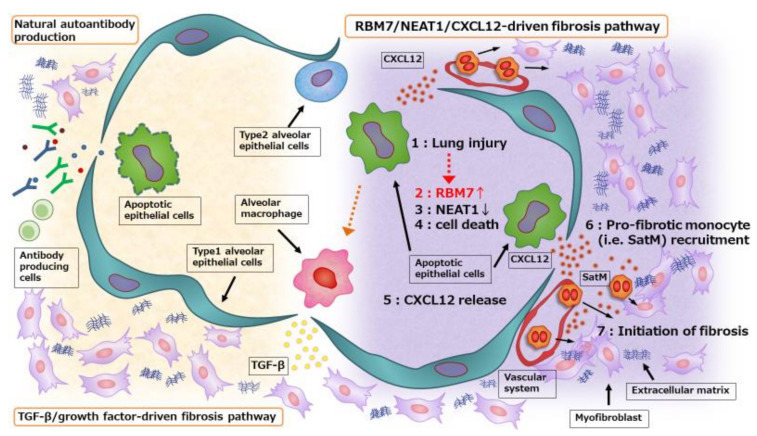
Schematic representation of fibrosis regulation by RBM7 via NEAT1 ncRNA decay in the lung. Following bleomycin (BLM) administration, RBM7 expression is selectively elevated in the fibrotic phase, and it leads to sustained apoptosis in the epithelium via regulating NEAT1 ncRNA decay. CXCL12 from apoptotic non-hematopoietic cells is critical for segregated-nucleus-containing atypical monocyte (SatM) migration followed by fibrosis initiation. These events, together with the conventional TGF-β/growth factor-driven fibrosis pathway, accelerates extracellular matrix (ECM) production by activated myofibroblasts. Also, apoptosis is the major source of autoantigens for natural autoantibodies, which can reflect the ongoing disease pathology and modify the disease course.

Here, we summarize recent insights into the pathogenesis and disease course of fibrotic lung disease in association with interactions between structured and non-structured cells triggered by cell death responses, and discuss their potential relevance to natural autoantibody production, which can modify the course of the original disease (Figure 1).

## 2. Cell Death in Structured Cells Initiates Fibrosis Development

Fibrosis, characterized by excessive ECM accumulation, is a progressive disease that eventually leads to failure of several critical organs. Following tissue damage, tissue repair mechanisms heal the resulting wound. However, when this process fails, a permanent fibrotic “scar” can be formed at the site of tissue injury, characterized by exacerbated accumulation of ECM components like hyaluronic acid, fibronectin, proteoglycans, and interstitial collagens. [13,14]. As a consequence, fibrogenesis is often defined as a dysregulated wound healing response accompanied by excessive accumulation of ECM [15].

Among experimental animal models, single intratracheal administration of the chemotherapeutic agent bleomycin (BLM) is widely used for the induction of lung fibrosis [16]. Although several histological characteristics of the BLM model, including epithelial cell injury followed by reactive hyperplasia that leads to interstitial fibrosis, are similar to those of IPF, the BLM model shows some marked differences from IPF, including partial reversibility and rapid onset and progression of fibrosis in response to severe injury to the lung. Despite several limitations, the BLM model continues to exist as a standard pulmonary fibrosis model for investigating disease pathogenesis and testing novel pharmaceutical compounds. Administration of BLM results in oxidative stress and acute lung injury accompanied by subsequent onset of pulmonary fibrosis. After intratracheal administration, BLM causes widespread oxidant-mediated DNA damage [17]. Bronchial epithelial cell death, including necroptosis and apoptosis, is observed as early as within a few hours [8,18]. Apoptotic death usually leads to immunologically silent responses, whereas necrotic or necroptotic death releases molecules like damage-associated molecular patterns (DAMPs) and pathogen-associated molecular patterns that promote inflammation [19,20]. Surprisingly and interestingly, the cell death responses after BLM administration are biphasic [8,18]. The initial cell death responses occur soon after BLM administration, peak at day 1, and subside thereafter. The subsequent cell death response begins to increase after the peak of inflammation and continues for more than 10 days. Previous reports suggested that cell death, regardless of induction by drug administration, infection, or physical trauma, can cause fibrosis through the release of endogenous ligands from dead cells and, importantly, that inhibition of this process by pan-caspase inhibitors can inhibit fibrosis development [21,22]. Indeed, several molecules involved in apoptosis regulation have been reported to be associated with lung fibrosis development, including increased alveolar epithelial cell Fas activation by FasL, upregulation of p53 expression and DNA damage induced by reactive oxygen species (ROS), decreased telomerase activity, and decreased Bcl-2 expression in lung epithelial cells [23]. In addition, transforming growth factor (TGF)-β, a central mediator of fibrogenesis, plays pro-apoptotic roles in structured cells [24], and the anti-fibrotic effects of pirfenidone are partially explained by reduced TGF-β expression [25]. In our previous study, we found that sustained apoptosis of structured cells, especially lung epithelial cells occurs in the fibrotic lung, and that this event is regulated by the novel fibrosis-related molecule RBM7 [8]. RBM7 is an RNA-binding protein and its physiological function has not been clearly understood. In 2011, RBM7 was identified as a component of a novel RNA exosome complex, termed the nuclear exosome targeting (NEXT) complex [26]. This RNA exosome complex is located in the nucleus and dedicated to RNA decay. RBM7 is selectively increased from the fibrotic phase in mice and humans, and genetic deletion of RBM7 suppresses fibrosis in multiple organs including the lung [8]. To determine the role of RBM7 in the fibrotic phase, we intravenously administered lung-specific siRNAs against RBM7 to wild-type mice during the fibrotic phase only. Of note, we found that repression of RBM7 alone in the fibrotic phase results in reduced fibrosis, suggesting that RBM7 in the fibrotic phase is critical for fibrosis development in the lung. RBM7 was shown to regulate the target RNA specificity of the NEXT complex and bind to U-rich stretches in RNAs, requiring a minimum of four pyrimidine nucleotides [27]. Previous studies revealed a role of the NEXT complex in the decay of long noncoding RNAs (lncRNAs) [28,29]. LncRNAs, which are longer than 200 nucleotides and not translated into proteins, have diverse functions, and the vast majority of individual mammalian genomes are transcribed into lncRNAs. Recent advances in lncRNAs research have revealed their involvement in multiple cellular contexts and biological processes [30]. These results suggest that RBM7 functions in essential cellular processes through RNA decay. Indeed, combined RNA immunoprecipitation and RNA-seq analyses showed that RBM7 regulates the degradation of specific sets of lncRNAs [8]. Among these, NEAT1 is associated and colocalized with RBM7, and RBM7 associates with NEAT1 through the RNA-binding motif in RBM7 and 7U sequences in NEAT1 RNA. Further, RBM7 promotes nuclear degradation of NEAT1 ncRNAs, and NEAT1 expression is increased in the RBM7-depleted condition. NEAT1 is transcribed from the multiple endocrine neoplasia locus. NEAT1 is retained in the nucleus and acts as a core molecule of paraspeckles [31]. Paraspeckles are recently identified subnuclear bodies that usually assemble co-transcriptionally at the NEAT1 transcription site. Various RNA-binding proteins are orchestrated around NEAT1 ncRNA, which functions as a molecular hub for paraspeckle formation [32]. It was recently shown that NEAT1 and paraspeckles function in diverse biological processes including DNA damage and apoptosis [33]. Therefore, RBM7 plays an essential role in regulating ncRNA decay and cell death, prior to the development of fibrosis. Taken together, excessive apoptosis of lung structured cells is the key cellular event leading to a dysregulated wound healing response and fibrosis.

## 3. Interaction between Structured and Non-Structured Cells in the Development of Fibrosis

The initial cell death response following BLM administration induces the release of large amounts of DAMPs that trigger the migration of non-structured cells such as Ly6c-positive immune cells, i.e., neutrophils and inflammatory monocytes, leading to a severe inflammatory response (inflammatory phase) [34]. Whether or not this severe inflammatory response is dispensable for fibrosis development is a widely debated issue and has pros and cons in the literature [35,36]. It has been reported that recruited inflammatory cells and lung resident alveolar macrophages produce large amounts of cytokines like interleukin (IL)-1β, TNF-α, and IL-13, and substantially contribute to the development of fibrosis [34]. However, depletion of the main inflammatory cells such as neutrophils and inflammatory monocytes with an anti-Gr1 antibody does not affect fibrosis development [37,38]. Furthermore, administration of anti-inflammatory drugs like dexamethasone does not inhibit fibrosis development [23,39]. Therefore, inflammation itself may not be an essential component of fibrosis development, and studies on fibrosis-specific cascades are recognized as important for understanding the pathogenesis of fibrosis onset.

Regarding the pathology of fibrosis, phagocytosis of apoptotic cells was reported to induce the production of pro-fibrotic cytokines, such as TGF-β, by macrophages [40,41,42]. TGF-β has been regarded as a key molecule for fibrosis regulation, and further induces the expression of connective tissue growth factor (CTGF) through a functional Smad3-binding site in the CTGF promoter [43]. Both TGF-β and CTGF are associated with fibroblast/myofibroblast accumulation and collagen deposition in fibrotic lesions [44]. In our previous study [8], we found that apoptotic lung structured cells, such as cleaved caspase 3-positive lung epithelial cells, produce large amounts of the chemokine CXCL12, which is required for the recruitment of non-structured immune cells like pro-fibrotic monocytes, i.e., fibrocytes and SatMs. Fibrocytes and SatMs are both derived from the monocyte lineage and are committed to fibrosis development. Fibrocytes were first described in 1994 as circulating bone marrow-derived cells that migrate to sites of injury [45]. Fibrocytes co-express hematopoietic and progenitor cell markers (CD45^+^ and CD34^+^), together with fibroblast markers (collagen1 and vimentin). Another typical characteristic of these cells is their spindle-shaped fibroblast-like morphology when adherent. SatMs have a bi-lobed segmented nuclear shape and many cytoplasmic granules. Their differentiation is licensed by CCAAT/enhancer-binding protein-β (CEBPβ). SatMs, which are derived from Ly6C^−^-FcεRI^+^ granulocyte/macrophage progenitors, have hybrid characteristics of both monocytes and granulocytes. CXCR4, the receptor for CXCL12 is highly expressed in fibrocytes and SatMs. Inhibition of the CXCL12/CXCR4 axis by neutralizing antibodies reduces the development of lung fibrosis through regulation of pro-fibrotic monocyte recruitment [8,46]. Therefore, CXCL12 is a critical chemokine for fibrosis development of the lung. Meanwhile, it is also essential for the tissue repair mechanism [47]. Thus, the CXCL12 balance is a key regulator of normal wound healing and fibrosis.

CXCL12 expression in lung tissue gradually increases from the early fibrotic phase, and CXCL12 is mainly expressed and produced by apoptotic lung structured cells. CXCL12, also known as cell growth-stimulating factor and stromal cell-derived factor-1, was initially identified in stromal cell lines from mice [48] and first characterized as a growth-stimulating factor in a B cell precursor clone [49]. CXCL12 is a member of the α-chemokine subfamily and CXCR4 is its specific receptor. CXCL12 and CXCR4 have been implicated in various developmental processes, including hematopoiesis, and regenerative processes [50,51,52]. Chemokines comprise a huge family of small cytokines (8−12 kDa) that bind to their corresponding G protein-coupled protein receptors and function in diverse cellular processes such as cell migration [53], tissue formation and regeneration, and recruitment of immune system cells to sites of inflammation and injury to increase chemokine gradients [54]. CXCR4 is internalized with CXCL12 via endocytosis by clathrin-coated pits dependent on epidermal growth factor receptor substrate 15 (Eps15) and RAS associated protein RAB5A (Rab5) [55]. After binding of CXCL12 to CXCR4, CXCR4 undergoes monoubiquitylation, endocytosis, and relocation to lysosomes for degradation [56]. The CXCL12-CXCR4 interaction activates several intracellular downstream signaling pathways such as phosphorylation cascades regulated by the SRC proto-oncogene, Src non-receptor tyrosine kinase, and AKT serine/threonine kinase that are involved in cell survival, proliferation, chemotaxis, and migration [57].

The CXCL12/CXCR4 axis is also implicated in several key processes for lung disorders including migration of immune cells to affected lesions. In asthma patients, stimulation of bone marrow by an allergen leads to reduced expression of CXCL12 and CXCR4, possibly leading to outflow of immune cells from bone marrow [58]. In a study on asthma patients, immunohistochemical staining of bronchial biopsy samples revealed that immunoreactivity of CXCL12 is highly correlated with vascularity [59]. Furthermore, in acute lung injury patients, resected samples from lung tissue show high expression levels of CXCL12 and its receptor CXCR4 is elevated in circulating granulocytes [60].

Previous studies examined the role of the CXCL12/CXCR4 system in lung fibrosis and demonstrated an essential role in fibrosis development, although the detailed mechanisms remain to be clarified. CXCL12 expression in the lung is increased at the late stage after BLM injury and CXCR4 expression in the lung accompanies this increase in CXCL12 expression [61,62]. Fibrocytes and SatMs both express CXCR4 and exert pro-fibrotic functions for trafficking to the lung via the CXCL12/CXCR4 axis. Inhibition of this axis with neutralizing antibodies against CXCL12 significantly reduces the development of lung fibrosis [46]. Several studies have investigated the effects of CXCR4 antagonistic chemical compounds in vitro and in vivo [63,64,65]. AMD3100, also called plerixafor, was first examined as an antagonist of CXCL12-CXCR4 signaling, and was shown to exhibit partial agonism at high concentrations [66]. One study using AMD3100 showed that it potently inhibits the recruitment of fibrocytes and lung fibrosis [64], while another study showed little to no effect of CXCR4 inhibition on BLM- or CCl_4_-induced lung and liver lung fibrosis [63]. In the latter report, the authors showed that treatment with AMD070, a related oral inhibitor of CXCR4, has a negligible impact on ECM deposition. Instead, they found that the inhibitor blocks the early inflammatory response and vascular leakage that contribute to mortality following BLM exposure. Meanwhile, the results of AMD3100 administration on lung fibrosis in other organs have also been controversial [67,68]. Recently, less cytotoxic, more potent, and stable CXCR4 inhibitors such as BL8040 [69] and LY2510924 [70] have been developed, and thus the effects of targeting the CXCL12/CXCR4 axis by CXCR4 inhibitors for the treatment of lung fibrosis should desirably be re-examined. Regarding human diseases, there is an increase in CXCL12 expression in lung epithelial cells and an elevated number of CXCR4-positive cells in IPF patients, suggesting that chronic injury in the epithelium induces the recruitment of CXCR4-positive cells such as SatMs and fibrocytes that may activate fibroblasts or act as sources for new fibroblasts [61]. Furthermore, anti-fibrotic agents like pirfenidone and nintedanib also reduce CXCL12 expression in lung structured cells [23,71].

TGF-β signaling induces and maintains CXCL12 signaling by elevating CXCL12 expression, and both TGF-β and CXCL12 induce CTGF expression in fibroblasts [72,73]. Therefore, crosstalk between the conventional TGF-β/growth factors-driven fibrosis pathway and the proposed novel RBM7-NEAT1-CXCL12 pathway would play integral roles in fibrosis development. Collectively, these signaling cascades result in robust fibrogenic responses that lead to ECM production by differentiated and activated α-smooth muscle actin-expressing myofibroblasts. Myofibroblasts are the primary cells responsible for enhanced ECM production, and are derived from a variety of sources including mesenchymal cells residing in the local environment, bone marrow-derived fibrocytes, and a process called epithelial mesenchymal transition, whereby epithelial cells transdifferentiate into fibroblast-like cells [34,74].

## 4. Natural Autoantibodies in Fibrotic Lung Disease

**Figure 2:**
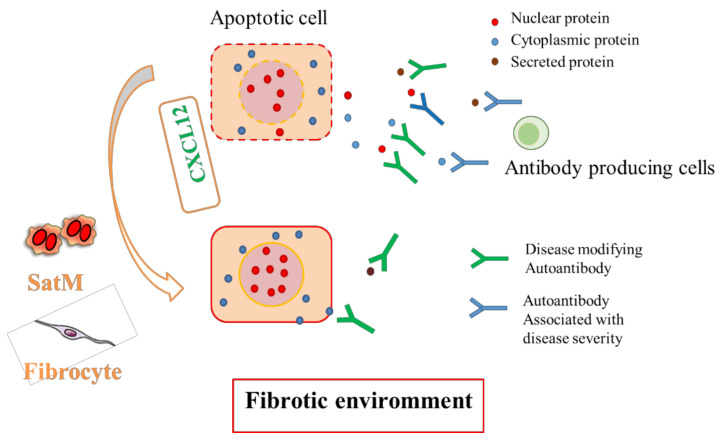
Proposed mechanisms for the associations between cell death responses and natural autoantibody production in fibrosis development. Sustained apoptosis in the fibrotic lung can induce natural autoantibody production, which can reflect the ongoing disease pathology and modify the disease course.

As discussed above, repetitive injury and sustained cell death of the epithelium in the fibrotic phase are crucial for lung fibrosis development. Since apoptosis elicits re-localization of several potential intracellular autoantigens to the cell surface and sometimes induces leakage of cellular contents, apoptotic cells act as an important source of self-antigens [75]. Recent studies have shown that sera from patients both with and without autoimmune diseases contain considerable amounts of IgG class autoantibodies that react with various self-antigens and differ among diseases [10]. These autoantibodies, designated natural autoantibodies, mostly react with intracellular proteins and lncRNAs [11], and are thought to have evolved as an adaptive mechanism for processes like clearance of debris from apoptotic cells, suggesting their promising utility as diagnostic and prognostic factors for various diseases. Thus, the environment in fibrotic lung diseases may accelerate increased production of fibrosis-specific natural autoantibodies, which can reflect the ongoing disease pathology and have the potential to modify the disease course (Figure 2). Indeed, in chronic fibrosing pulmonary diseases, previous studies have demonstrated correlations between autoantibodies and disease prognosis [76].

Currently, antifibrotic drugs such as pirfenidone and nintedanib are widely used in clinical settings, and the precise classification of chronic fibrotic lung disease is of great importance for the selection of appropriate patients who will gain beneficial effects from these drugs. Chronic lung fibrotic diseases mostly comprise IPF, idiopathic nonspecific interstitial pneumonia, and other unclassifiable chronic fibrosing interstitial pneumonias [1]. For clustering of patients with chronic fibrotic lung diseases without definite diagnoses, the usefulness of circulating autoantibodies has been reported [77]. For example, chronic fibrotic lung disease patients positive for anti-aminoacyl-tRNA synthetase (ARS) autoantibodies were reported to show good responses to immunosuppressive therapy and have a better prognosis than patients with IPF [78]. Meanwhile, patients positive for anti-melanoma differentiation-associated gene 5 (MDA5) autoantibodies tend to have progressive fibrotic interstitial lung disease with a poor prognosis [79]. Since no studies have directly proven the pathogenesis of these autoantibodies [80,81], they are thought to represent natural autoantibodies in chronic fibrotic lung diseases. Therefore, natural autoantibodies would be useful for investigating the pathogenesis and disease course of fibrotic lung diseases. We previously reported the utility of protein array analyses for discovering novel autoantibody biomarkers and gaining insights into disease pathogenesis [82]. Protein arrays consist of panels containing more than 8000 peptides and proteins, including known and candidate autoantigens. Through comprehensive protein array analyses, we identified IPF-specific natural autoantibody subsets that recognize distinct sets of antigens in IPF [12,82]. These findings are important because if the specific sets of antigens can reflect the ongoing pathophysiological processes that are upregulated in fibrotic lesions, we can determine the molecular events that occur in the disease environment through examination of patient sera [12]. Sera from IPF patients react with molecules associated with TGF-β and fibroblast activation (transgelin 2 [83], transgelin 3 [84], LIM domain-binding protein 2 [85], HLA complex P5 [86], PHGDH [87], NAT6 [88], CDK9 [89], SEPT4 [90]), cell death regulation (14-3-3 protein zeta/delta [91], Trefoil factor 2 protein [92], RAS-like family 11 member B [93], MRPS11 [94], RSU1 [95], PLCG2 [96], IFI44L [97], YTHDF2 [98], AMOTL2 [99], ROGDI [100]), and airway clearance (sperm flagellar 1 [101], cilia and flagella associated protein 410 [102], t-complex 10 like [103]) (Table 1). Natural autoantibodies are thought to reflect the ongoing disease environment [10,11]. Therefore, some antigens for natural antibodies are key molecules for disease development and progression. Future research to broaden our knowledge about IPF-specific natural autoantibody subsets and their related antigens will reveal the essential physiological processes and mechanisms involved in fibrosis onset.

## 5. Conclusions

Fibrous connective tissue formation is the hallmark of fibrosis, which is thought to result from an abnormal wound healing process upon repetitive injury. Fibrosis is a chronic progressive disease associated with significant morbidity and mortality. Recent investigations have revealed several key events and provided insights into the pathophysiology of fibrosis onset. Currently, fibrosis is no longer believed to result from chronic inflammation alone, but instead from repetitive injury to the epithelium, and the subsequent cell death is considered the important triggering event of fibrosis. Repetitive injury leads to a vast amount of apoptosis in the epithelium and induces proliferation of activated myofibroblasts, followed by the formation of a fibrotic lesion. Interactions between structured cells and non-structured cells are critical for fibrosis development by bridging these epithelium-myofibroblast cascades. CXCL12, an essential chemokine for wound healing and tissue homeostasis, is also important for lung fibrosis development by regulating the migration of pro-fibrotic monocytes such as fibrocytes and SatMs. In injured tissue, RBM7 expression is selectively increased in the fibrotic phase and sustained apoptosis occurs after NEAT1 degradation. This leads to increased production of CXCL12, resulting in SatM recruitment and fibrosis development. In addition, evidence suggests that crosstalk between the conventional TGF-β/growth factor-driven fibrosis pathway and the proposed novel RBM7-NEAT1-CXCL12 pathway reinforces the pathways and plays an integral role in fibrosis development. Furthermore, apoptosis is a major source of self-antigens for natural autoantibodies. Sustained apoptosis in the fibrotic lung also induces natural autoantibody production, which can reflect the ongoing disease pathology and modify the disease course. Future studies will clarify the complex crosstalk and diverse mechanisms among immune and non-immune cells bridging innate and adaptive immunity.

## Figures and Tables

**Table 1:** Related signaling pathways of antigens for idiopathic pulmonary fibrosis (IPF)-specific natural autoantibodies enriched by protein array analysis [82].

Signalling Pathways	Antigens for IPF Specific Natural AutoAbs
TGF-β and fibroblast activation	transgelin 2 [83], transgelin 3 [84], LIM domain-binding protein 2 [85], HLA complex P5 [86], PHGDH [87], NAT6 [88], CDK9 [89], SEPT4 [90]
cell death regulation	14-3-3 protein zeta/delta [91], Trefoil factor 2 protein [92], RAS-like family 11 member B [93], MRPS11 [94], RSU1 [95], PLCG2 [96], IFI44L [97], YTHDF2 [98], AMOTL2 [99], ROGDI [100]
airway clearance	sperm flagellar 1 [101], cilia and flagella associated protein 410 [102], t-complex 10 like [103]
